# Focal Segmental Glomerulosclerosis With Superimposed Infection‐Related Glomerulonephritis in a Diabetic Patient: A Case of Rapid Renal Decline

**DOI:** 10.1002/ccr3.9593

**Published:** 2024-11-20

**Authors:** Mahyar Toofantabrizi, Sena Armagan, Suchitra Shrestha, Rahul Singla, Saurab Karki, Ajeet Singh, Amit Varma, Mahendra Pratap Singh, Sanjit Sah, Amogh Verma

**Affiliations:** ^1^ MedStar Franklin Square Medical Center Baltimore Maryland USA; ^2^ Department of Emergency Medicine Military Hospital Itahari Nepal; ^3^ Department of Internal Medicine Dow University of Health Sciences Karachi Pakistan; ^4^ Department of General Medicine Graphic Era (Deemed to be University) Clement Town Dehradun Dehradun India; ^5^ Center for Global Health Research, Saveetha Medical College and Hospital, Saveetha Institute of Medical and Technical Sciences Saveetha University Chennai India; ^6^ Evidence for Policy and Learning Global Center for Evidence Synthesis Chandigarh India; ^7^ Dr. D. Y. Patil Medical College, Hospital and Research Centre Dr. D. Y. Patil Vidyapeeth Pune Maharashtra India; ^8^ Dr. D.Y. Patil Dental College and Hospital Dr. D.Y. Patil Vidyapeeth Pune Maharashtra India; ^9^ SR Sanjeevani Hospital Kalyanpur Siraha Nepal

**Keywords:** diabetic nephropathy, end‐stage renal disease, focal segmental glomerulosclerosis (FSGS), infection‐related glomerulonephritis (IRGN), rapid renal decline

**Summary**

- Early and accurate differentiation of the contributing pathologies is crucial to guide therapy in patients with rapidly worsening renal function and a combination of focal segmental glomerulosclerosis (FSGS) and infection‐related glomerulonephritis (IRGN), especially those with underlying diabetes and morbid obesity.
- Immunosuppressive treatments for FSGS must be judiciously balanced with infection control strategies in the presence of IRGN, as inappropriate management can accelerate progression to ESRD.
- Multidisciplinary collaboration and timely and precise histopathological assessment are essential for optimizing outcomes and preventing irreversible kidney damage in high‐risk patients.

## 1 Introduction

Focal segmental glomerulosclerosis (FSGS) is a significant cause of nephrotic syndrome, particularly in adults [1, 2]. It is characterized by segmental scarring of the glomeruli, leading to proteinuria and progressive loss of kidney function [3]. FSGS can arise primarily (idiopathic) or secondarily due to underlying conditions such as obesity, diabetes, infections, or nephrotoxic drugs [4].

In the context of diabetes and obesity, secondary FSGS often develops due to metabolic stress on the glomeruli [5, 6]. Obesity increases glomerular hyperfiltration and hypertrophy, leading to increased glomerular capillary wall tension and subsequent podocyte injury [7]. Hyperglycemia in diabetes induces oxidative stress and the formation of advanced glycation end‐products, which damage the glomerular filtration barrier [8]. These metabolic derangements accelerate renal decline by promoting glomerulosclerosis and proteinuria.

Infection‐related glomerulonephritis (IRGN) is an immune‐mediated condition that develops in response to systemic infections, typically involving bacteria such as *Staphylococcus* or *Streptococcus* [9, 10]. The formation of immune complexes following an infection leads to glomerular inflammation and injury [11]. Although post‐streptococcal glomerulonephritis (PSGN) is well‐recognized in children, IRGN in adults often presents differently [12]. Adults are more likely to have underlying comorbidities such as diabetes, which impair immune function and promote persistent infections [13]. Recovery of renal function is less common in adults with IRGN due to factors like delayed diagnosis, ongoing infections, and the presence of other renal pathologies like diabetic nephropathy [14].

This case report presents a rare case of IRGN superimposed on FSGS in a middle‐aged patient with morbid obesity, diabetes, and chronic skin infections. The coexistence of these two pathologies significantly complicates both the diagnostic process and the therapeutic strategies. Understanding the interplay between FSGS and IRGN is crucial for effective management, as each condition may necessitate distinct treatment approaches, and the overlap can hasten progression to end‐stage renal disease (ESRD) [15, 16].

## 2 Case Presentation

A 48‐year‐old Caucasian male with a body mass index (BMI) of 56, a history of hypertension, insulin‐dependent type 2 diabetes mellitus, and chronic lower extremity lymphedema presented with generalized weakness and persistent infected wounds on the left leg. The patient had been hospitalized 3 months prior to worsening bilateral leg edema and multiple superficial wounds. At that time, his creatinine level had increased to 3.15 mg/dL from the baseline of 1.2 mg/dL, and he received antibiotics with local wound care. On admission, the patient's vital signs were stable, except for elevated blood pressure. Physical examination revealed significant bilateral lower extremity edema with multiple infected wounds and severe stasis dermatitis.

## 3 Methods (Differential Diagnosis, Investigation, and Treatment)

Initial laboratory results showed acute kidney injury (AKI) superimposed on chronic kidney disease (CKD), with a creatinine level of 8.17 mg/dL (baseline creatinine 3 months prior was 3.15 mg/dL), blood urea nitrogen (BUN) of 96 mg/dL, and severely reduced glomerular filtration rate (GFR) of 7 mL/min/1.73 m^2^. Creatinine progression over the past 3 months indicated a rapid decline in renal function. Immunoglobulin G (IgG) was elevated to 2329 mg/dL, with a normal kappa/lambda ratio. Complement levels revealed decreased C3 levels (35 mg/dL), and proteinuria was significant at > 300 mg/dL. These laboratory findings are summarized in Table 1.

**TABLE 1:** Laboratory findings reflecting renal impairment and immune dysregulation.

Lab	Result	Normal range	Interpretation
Blood urea nitrogen (BUN)	96 mg/dL	9–23 mg/dL	Elevated
Creatinine	8.17 mg/dL	0.6–1.1 mg/dL	Elevated
Glomerular filtration rate (GFR)	7 mL/min/1.73 m^2^	> 60 mL/min/1.73 m^2^	Severely decreased
Immunoglobulin G (IgG)	2329 mg/dL	650–1600 mg/dL	Elevated
Serum free kappa	491 mg/L	3.30–19.40 mg/L	Elevated
Serum free lambda	424 mg/L	5.71–26.30 mg/L	Elevated
Kappa/Lambda ratio	1.16	0.26–1.65	Normal
C3 complement	35 mg/dL	90–170 mg/dL	Decreased
Proteinuria	> 300 mg/dL	< 30 mg/dL	Elevated
Alkaline phosphatase	193 units/L	46–116 units/L	Elevated
Body Mass Index (BMI)	56	20–25	Elevated (indicating obesity)

Given the patient's clinical presentation and laboratory findings, the differential diagnosis included acute exacerbation of diabetic nephropathy, FSGS, IRGN, and autoimmune‐mediated glomerulonephritis, such as lupus nephritis or anti‐neutrophil cytoplasmic antibody (ANCA)‐associated vasculitis. Autoimmune serologies, including antinuclear antibodies (ANA), ANCA, anti‐glomerular basement membrane (anti‐GBM) antibodies, and complements, were evaluated to rule out autoimmune causes.

**FIGURE 1:**
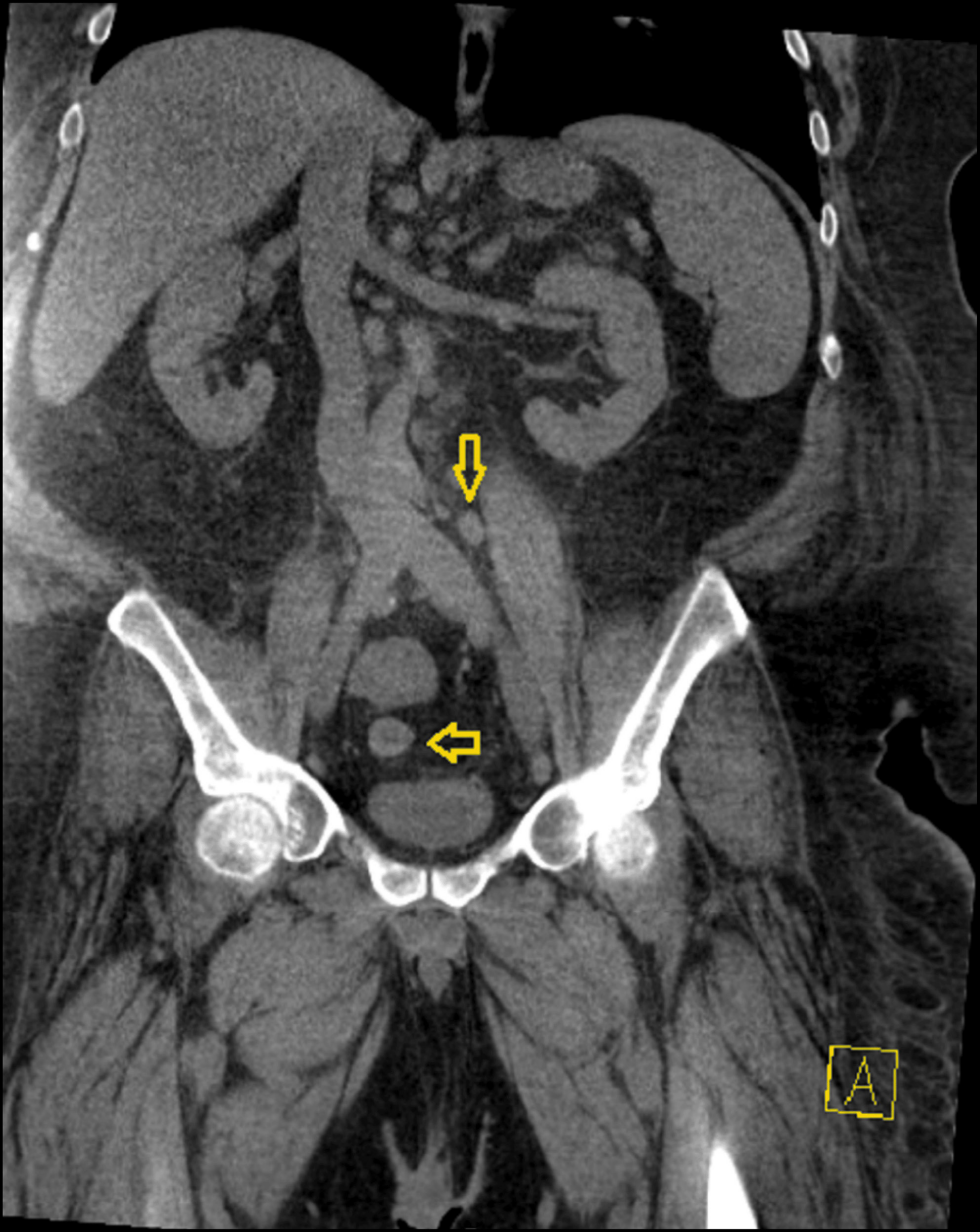
Retroperitoneal lymphadenopathy. Computed tomography (CT) scan showing mild retroperitoneal lymphadenopathy, likely indicating reactive lymphadenopathy due to chronic infection or immune activation. The yellow arrow points to the enlarged retroperitoneal lymph nodes.

**FIGURE 2:**
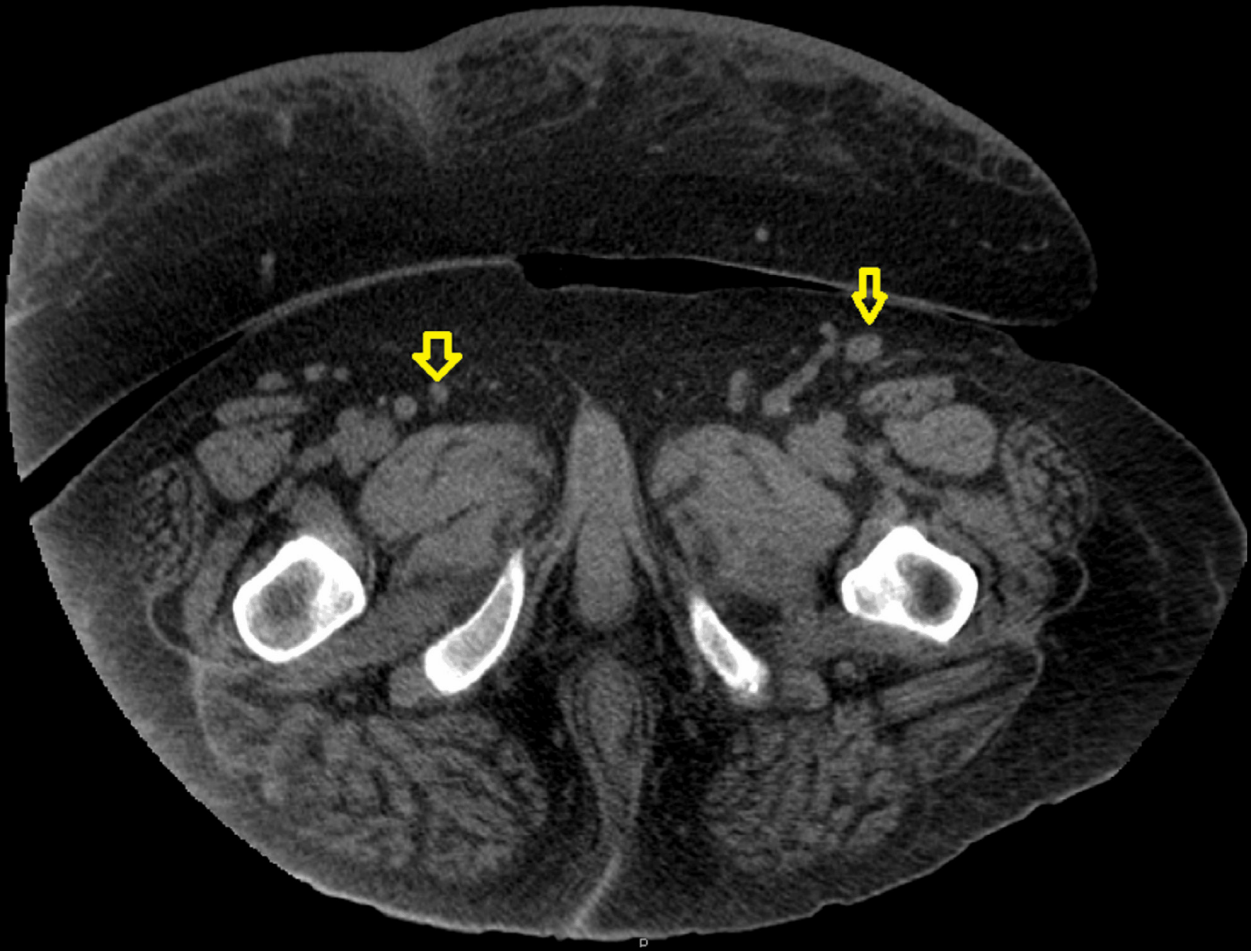
Inguinal lymphadenopathy. Computed tomography (CT) scan revealing bilateral inguinal lymphadenopathy, with the left side being more pronounced (measuring up to 2.1 cm) than the right. The yellow arrow indicates the enlarged inguinal lymph nodes, suggesting reactive changes.

**FIGURE 3:**
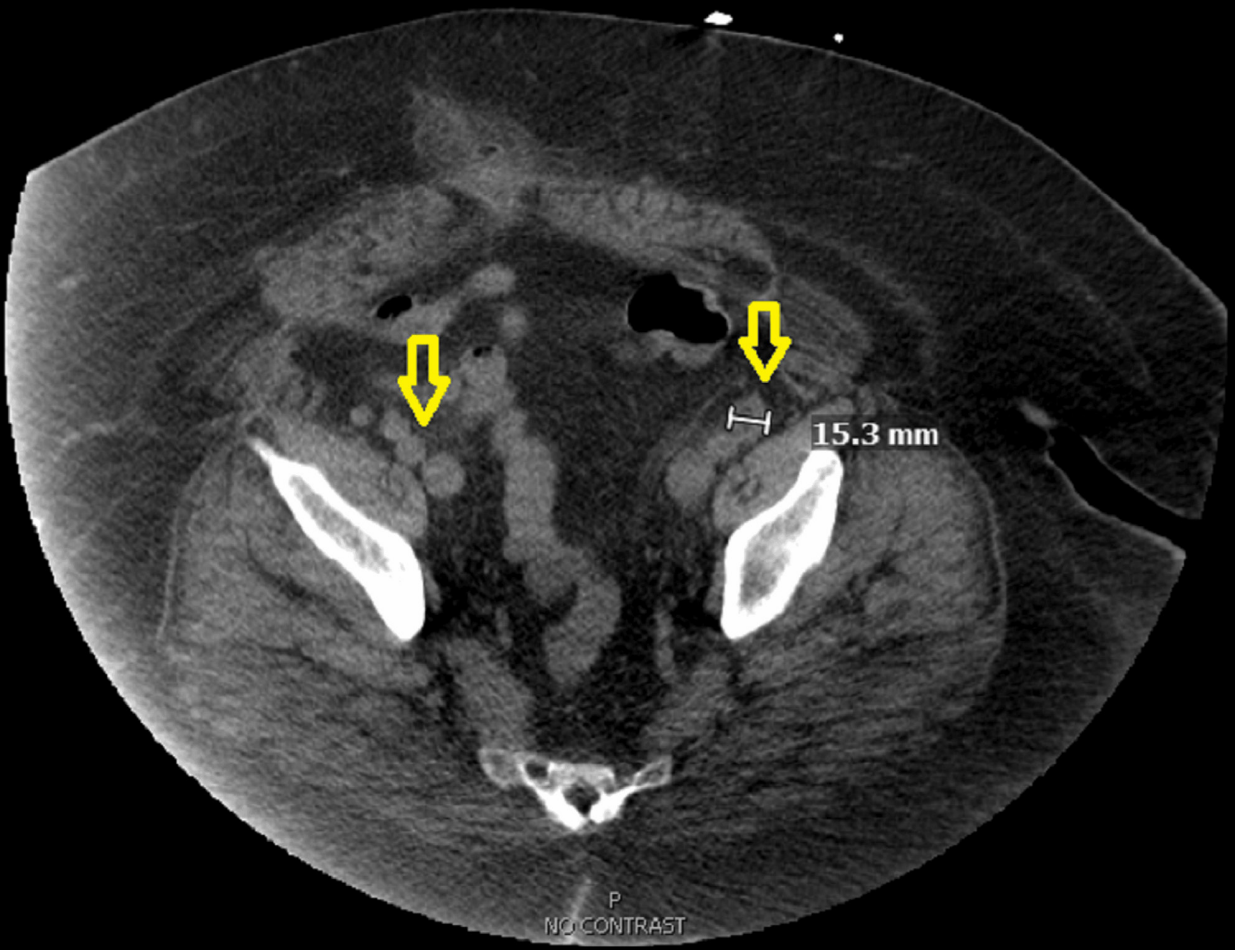
Pelvic lymphadenopathy. Computed tomography (CT) scan showing bilateral external iliac chain lymphadenopathy, with the left external iliac chain measuring up to 1.5 cm. The yellow arrow indicates the enlarged external iliac lymph nodes, consistent with reactive lymphadenopathy.

Abdominal computed tomography (CT) revealed retroperitoneal, pelvic, and inguinal lymphadenopathy, ranging from 15 to 21 mm, as shown in Figures 1, 2, 3. Lymphadenopathy was considered reactive, potentially due to chronic infections or immune activation. A renal biopsy was performed to ascertain the underlying pathology. Histopathological examination revealed FSGS with nodular diabetic glomerulosclerosis, and features consistent with IRGN. Immunofluorescence microscopy showed fine granular IgG and C3 staining in a “starry sky” pattern along the mesangial and capillary walls, consistent with the immune complex deposition characteristic of IRGN (Figures S1–, S3).

Given the active infection and evidence of IRGN, immunosuppressive therapy was avoided to prevent exacerbation of infection. Management focused on infection control with appropriate antibiotics, wound care, and supportive measures for renal function. The patient's renal function continued to decline, necessitating hemodialysis three times a week.

## 4 Results (Outcomes and Follow‐Up)

The patient was diagnosed with FSGS superimposed on IRGN based on the clinical, biochemical, and histopathological findings. The rapid progression of renal dysfunction has been attributed to the combined effects of FSGS and IRGN, exacerbated by uncontrolled diabetes and morbid obesity. These comorbidities likely contributed to both the onset of FSGS through metabolic and hemodynamic stress on the glomeruli and the exacerbation of IRGN by impairing immune function and promoting persistent infections.

Hemodialysis was initiated due to the rapid decline in renal function and severe uremia. Given the active skin infections and the presence of IRGN, immunosuppressive therapy was contraindicated. The patient was managed with antibiotics, aggressive wound care, and optimization of the diabetes and hypertension. The patient was discharged to an outpatient dialysis center for ongoing management.

Long‐term management included strict glycemic control, weight management strategies, and the treatment of chronic infections to minimize further renal injury. Despite these measures, the prognosis remained guarded due to the advanced stage of CKD and the presence of multiple high‐risk factors for poor renal recovery.

## 5 Discussion

The management of renal disease becomes particularly challenging when patients present with multiple overlapping pathologies, as observed in this case of FSGS superimposed with IRGN. The diagnostic complexity arises from the fact that both conditions, although distinct, share similar clinical features, such as proteinuria, progressive renal dysfunction, and a predisposition to CKD (Table 2). However, the underlying pathophysiological mechanisms driving each condition require separate considerations for diagnosis and management.

**TABLE 2:** Comparison of clinical features and treatment strategies of FSGS and IRGN in the patient.

Feature	FSGS	IRGN
Etiology in patient	Secondary to obesity and diabetes, leading to metabolic stress on glomeruli	Triggered by chronic skin infection leading to immune complex formation
Pathophysiology	Podocyte injury and segmental glomerular scarring due to hemodynamic stress and hyperfiltration	Immune complex deposition and complement activation causing glomerular inflammation
Clinical presentation	Proteinuria, progressive renal dysfunction	Hematuria, decreased complement levels, acute renal impairment
Histopathological findings	Segmental sclerosis, foot process effacement on electron microscopy	“Starry sky” pattern of IgG and C3 deposits on immunofluorescence, cellular crescents with fibrinoid necrosis
Treatment strategies	Potential use of immunosuppressive therapy, ACE inhibitors/ARBs, SGLT2 inhibitors, weight management	Antibiotic therapy to control infection, supportive renal care, avoid immunosuppression during active infection
Management in patient	Immunosuppressive therapy avoided due to active infection; focus on controlling diabetes, hypertension, and weight	Antibiotics and wound care prioritized; immunosuppression contraindicated
Prognosis	Risk of progression to ESRD if not adequately managed	Variable; poorer renal recovery in adults with comorbidities

Abbreviations: ACE, angiotensin‐converting enzyme; ARBs, angiotensin II receptor blockers; C3, complement component 3; ESRD, end‐stage renal disease; FSGS, focal segmental glomerulosclerosis; IgG, immunoglobulin G; IRGN, infection‐related glomerulonephritis; SGLT2, sodium‐glucose cotransporter‐2.

FSGS in this patient was likely secondary, driven by metabolic derangements from obesity and type 2 diabetes mellitus. Both conditions are well‐established risk factors for glomerular injury [17, 18]. In obesity‐related FSGS, increased body mass places excessive hemodynamic stress on the glomeruli, resulting in compensatory hyperfiltration and, eventually, segmental sclerosis [19, 20]. Diabetes further compounds this by inducing hyperglycemia‐induced damage to the glomerular filtration barrier, leading to proteinuria and progressive glomerular sclerosis [21, 22].

On the other hand, IRGN is an immune‐mediated glomerulonephritis triggered by an infection, with immune complexes depositing in the glomeruli and activating the complement system. The patient's history of chronic skin infection likely served as a precipitating factor for IRGN. Although PSGN is more common in children, the increasing incidence of IRGN in adults, particularly in those with diabetes or chronic infections, reflects a shift in the disease pattern [23]. In adults, the glomerular recovery following IRGN is often incomplete, especially when coupled with underlying glomerular pathologies like FSGS [24].

Comorbidities such as diabetes and obesity negatively impact the prognosis of IRGN in adults [25]. Diabetes impairs immune function, leading to prolonged or recurrent infections, and contributes to ongoing immune complex formation [26]. Obesity exacerbates glomerular hyperfiltration and promotes chronic inflammation, further damaging the kidneys [27]. Consequently, adults with IRGN and underlying comorbidities have poorer renal recovery rates compared to pediatric cases [28, 29].

In cases where both FSGS and IRGN coexist, kidney biopsy is crucial to distinguish between the two entities and to assess the degree of immune‐mediated damage [30]. In this patient, the biopsy revealed characteristic features of both conditions: segmental sclerosis associated with FSGS and immune complex deposition seen in IRGN. Immunofluorescence microscopy played a key role in confirming IRGN, with the “starry sky” pattern of IgG and C3 staining, which is highly indicative of immune complex glomerulonephritis [31]. Early diagnostic interventions, particularly kidney biopsies, are essential to differentiate between overlapping renal pathologies and to guide appropriate management.

The coexistence of these two renal pathologies presents a clinical challenge as their treatments may be conflicting. Although immunosuppressive therapy is often considered for FSGS, it is contraindicated in active infections such as IRGN, where immunosuppression could worsen the underlying infection [32]. Hence, the treatment strategy must prioritize infection control and supportive renal management in the acute phase of IRGN, while managing the long‐term implications of FSGS [33]. In this case, immunosuppressive therapy was avoided to prevent exacerbation of the chronic skin infection.

The prognosis of patients with overlapping FSGS and IRGN is generally poor, with a high likelihood of progression to ESRD, especially when diabetes and obesity are present [34, 35]. The patient's rapid decline in renal function, leading to the initiation of hemodialysis within days of presentation, highlights the severity of the condition. Long‐term management focuses on mitigating further renal damage by controlling contributing factors, including optimizing glycemic control, weight reduction, and managing hypertension.

For FSGS, angiotensin‐converting enzyme inhibitors (ACEIs) or angiotensin II receptor blockers (ARBs) are essential to reduce proteinuria and slow disease progression [36]. In patients where secondary FSGS is driven by metabolic stress, lifestyle interventions—such as weight management and dietary modifications—are critical. However, these interventions must be balanced with the patient's overall health status, especially in those with severe comorbidities such as morbid obesity. Given the increasing incidence of secondary FSGS due to metabolic diseases such as diabetes and obesity, there is a growing need for targeted therapies that address the underlying metabolic and hemodynamic stressors [37]. Novel therapies, including endothelin receptor antagonists, sodium‐glucose cotransporter‐2 (SGLT2) inhibitors, and APOL1‐targeted therapies, are being explored for their potential to reduce proteinuria and protect against glomerular damage in patients with FSGS and diabetic nephropathy [38, 39, 40]. SGLT2 inhibitors, in particular, have been shown to slow the progression of CKD in diabetic nephropathy and may also offer protective effects on podocytes, benefiting patients with FSGS [41, 42]. However, their role in active IRGN remains to be clarified.

Management of IRGN primarily involves eradicating the underlying infection to prevent further immune‐mediated damage. Antibiotic therapy targeting the causative pathogen is essential alongside supportive care to manage acute renal impairment. In severe cases of IRGN, where there is significant immune complex deposition and persistent inflammation, short courses of immunosuppressive therapy may be considered once infection is controlled. This approach must be used with caution because of the risk of secondary infections and other adverse effects. For instance, high‐dose steroid use in COVID‐19 patients led to increased antimicrobial use, although not necessarily higher infection rates [43].

Future research on IRGN should focus on understanding the immune mechanisms driving disease progression and developing targeted therapies to prevent immune complex deposition while preserving the body's ability to fight infection. Advances in diagnostics, particularly noninvasive biomarkers, could help in the early detection and differentiation of IRGN from other glomerular diseases, enabling more personalized treatment approaches. Further research should also aim to identify biomarkers that can differentiate between FSGS and IRGN in complex cases, facilitating earlier and more precise diagnosis. Novel therapeutic approaches that address both FSGS and IRGN without compromising infection control are needed. Clinical trials investigating the efficacy of targeted therapies, such as endothelin receptor antagonists or immunomodulatory agents, with minimal immunosuppressive effects, could provide new treatment options for patients with dual diagnoses.

## 6 Conclusion

This case emphasizes the complexity of managing FSGS superimposed with IRGN in patients with multiple comorbidities such as obesity and diabetes. Early diagnosis, appropriate biopsy‐based investigations, and a multidisciplinary approach to management are critical for preventing the rapid progression to ESRD. As the prevalence of metabolic diseases continues to increase, clinicians must remain vigilant in recognizing secondary forms of FSGS, particularly in patients with active infections. Continued research on novel therapeutic options and a deeper understanding of the immune processes involved in IRGN are essential for improving the outcomes in these challenging cases.

## Author Contributions

**Mahyar Toofantabrizi:** conceptualization, project administration, writing – original draft, writing – review and editing. **Sena Armagan:** conceptualization, project administration, writing – original draft, writing – review and editing. **Suchitra Shrestha:** conceptualization, project administration, writing – original draft, writing – review and editing. **Rahul Singla:** conceptualization, project administration, writing – original draft, writing – review and editing. **Saurab Karki:** writing – original draft, writing – review and editing. **Ajeet Singh:** writing – original draft, writing – review and editing. **Amit Varma:** writing – original draft, writing – review and editing. **Mahendra Pratap Singh:** writing – original draft, writing – review and editing. **Sanjit Sah:** writing – original draft, writing – review and editing. **Amogh Verma:** validation, writing – original draft, writing – review and editing.

## Ethics Statement

The authors have nothing to report.

## Consent

Written informed consent was obtained from the patient according to the journal's policies.

## Conflicts of Interest

The authors declare no conflicts of interest.

## Supporting information


**Figure S1.** Cellular Crescents with Fibrinoid Necrosis Under Light Microscopy. Kidney biopsy specimen viewed under light microscopy, showing cellular crescents with fibrinoid necrosis, a feature associated with severe glomerular injury in IRGN.

**Figure S2.** Fine Granular Mesangial and Capillary Staining for IgG and C3 with Immunofluorescence. Immunofluorescence microscopy demonstrating fine granular staining of IgG and C3 along the mesangial areas and capillary walls in a “starry sky” pattern, consistent with immune complex deposition in IRGN.

**Figure S3.** Diffuse Glomerular Basement Membrane Thickening and Severe Mesangial Matrix Expansion in a Nodular Pattern on Electron Microscopy. Electron microscopy image showing diffuse thickening of the glomerular basement membranes and severe mesangial matrix expansion in a nodular pattern, characteristic of diabetic glomerulosclerosis. Complete foot process effacement of podocytes is also observed, indicating severe podocyte injury consistent with FSGS.

## Data Availability

All relevant data, including figures and additional information, are provided within the manuscript. Any further details can be obtained by contacting the corresponding author.
