# Supplementary figures and images for: Focal Segmental Glomerulosclerosis With Superimposed Infection‐Related Glomerulonephritis in a Diabetic Patient: A Case of Rapid Renal Decline
Source: Clin Case Rep. 2024 Nov 20;12(11):e9593. doi: 10.1002/ccr3.9593 (PMC11578678; doi:10.1002/ccr3.9593)

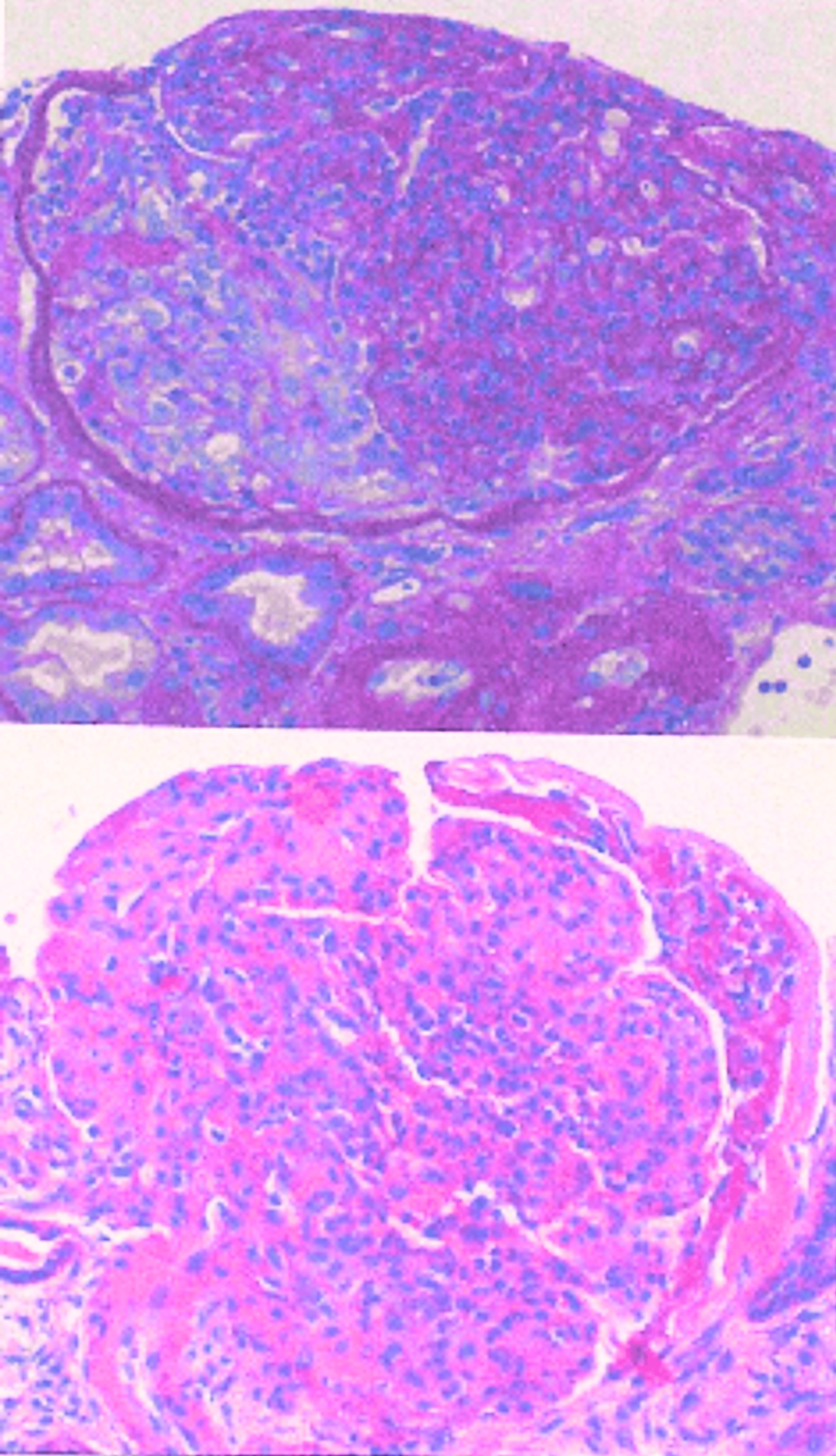

Supplement: Supplementary file 1 — Figure S1. Cellular Crescents with Fibrinoid Necrosis Under Light Microscopy. Kidney biopsy specimen viewed under light microscopy, showing cellular crescents with fibrinoid necrosis, a feature associated with severe glomerular injury in IRGN. [file CCR3-12-e9593-s003.jpg]

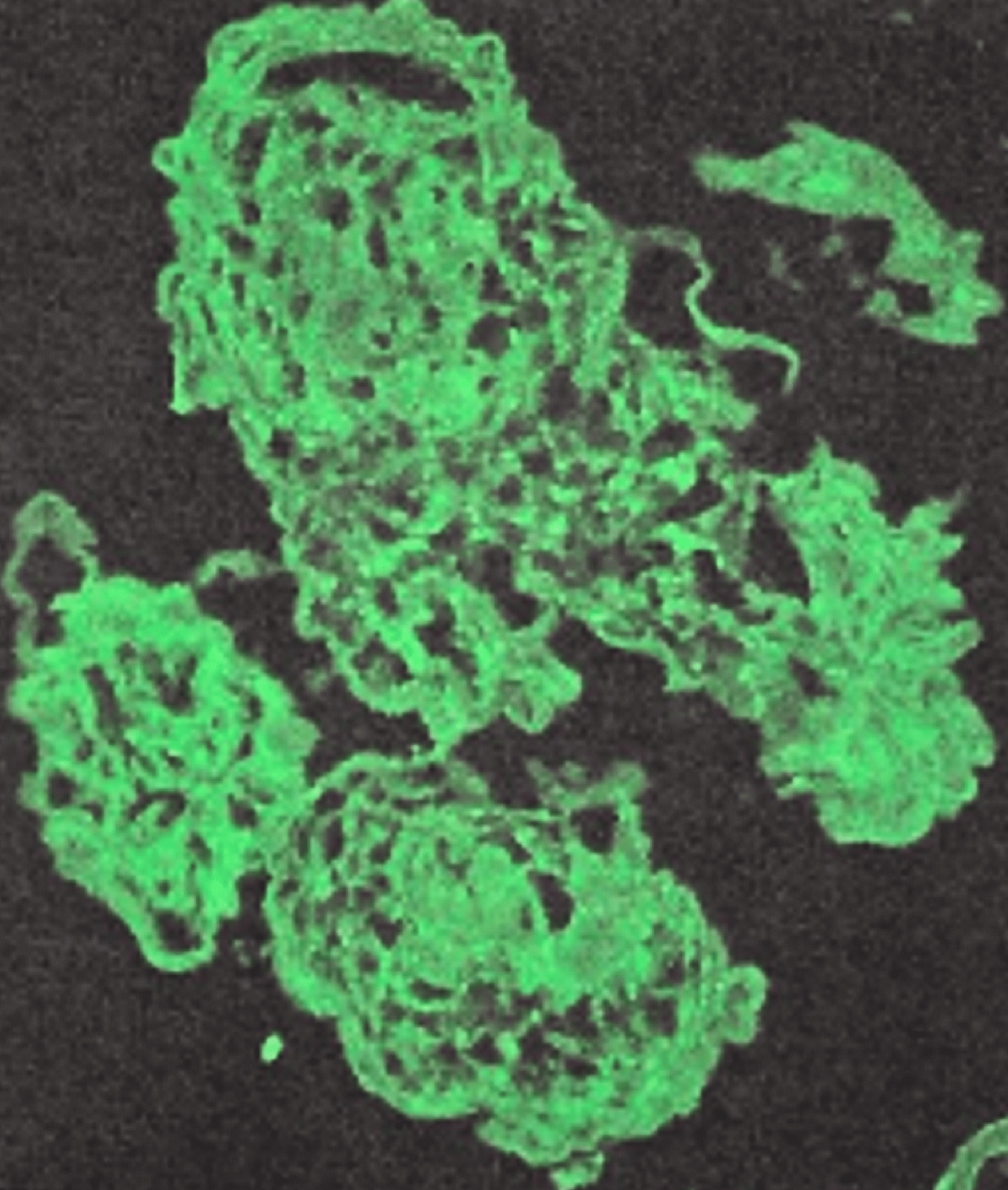

Supplement: Supplementary file 2 — Figure S2. Fine Granular Mesangial and Capillary Staining for IgG and C3 with Immunofluorescence. Immunofluorescence microscopy demonstrating fine granular staining of IgG and C3 along the mesangial areas and capillary walls in a “starry sky” pattern, consistent with immune complex deposition in IRGN. [file CCR3-12-e9593-s002.jpg]

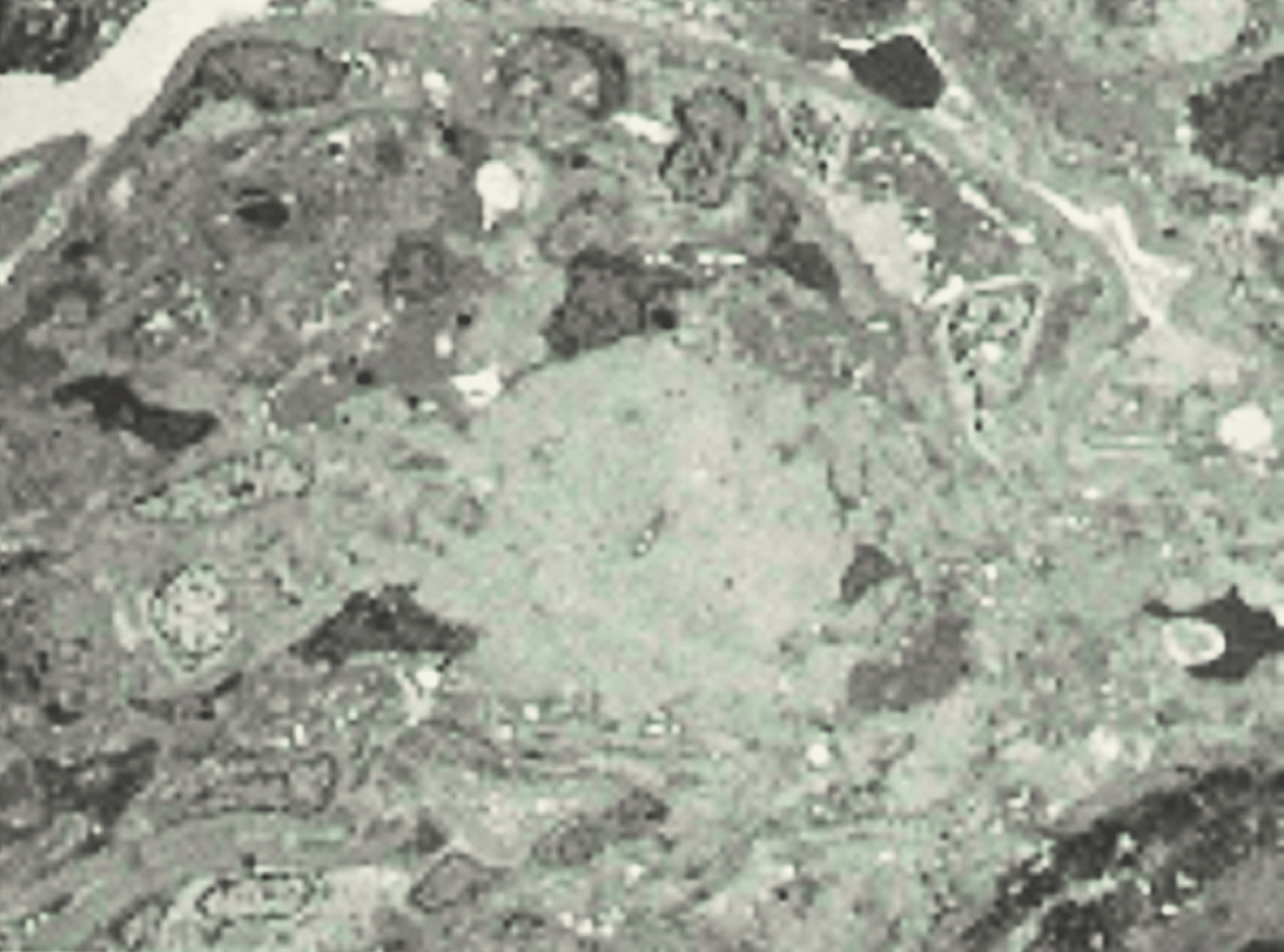

Supplement: Supplementary file 3 — Figure S3. Diffuse Glomerular Basement Membrane Thickening and Severe Mesangial Matrix Expansion in a Nodular Pattern on Electron Microscopy. Electron microscopy image showing diffuse thickening of the glomerular basement membranes and severe mesangial matrix expansion in a nodular pattern, characteristic of diabetic glomerulosclerosis. Complete foot process effacement of podocytes is also observed, indicating severe podocyte injury consistent with FSGS. [file CCR3-12-e9593-s001.jpg]
